# Depressive symptoms are doubled in older British South Asian and Black Caribbean people compared with Europeans: associations with excess co-morbidity and socioeconomic disadvantage

**DOI:** 10.1017/S0033291714002967

**Published:** 2015-02-13

**Authors:** E. D. Williams, T. Tillin, M. Richards, C. Tuson, N. Chaturvedi, A. D. Hughes, R. Stewart

**Affiliations:** 1 International Centre for Circulatory Health, Imperial College London, UK; 2 Institute of Cardiovascular Science, University College London, UK; 3 MRC Unit for Lifelong Health and Ageing, University College London, UK; 4 King's College London (Institute of Psychiatry), London, UK

**Keywords:** Depression, ethnic group differences, explanatory factors, prevalence

## Abstract

**Background:**

Despite elevated risk profiles for depression among South Asian and Black Caribbean people in the UK, prevalences of late-life depressive symptoms across the UK's three major ethnic groups have not been well characterized.

**Method:**

Data were collected at baseline and 20-year follow-up from 632 European, 476 South Asian and 181 Black Caribbean men and women (aged 58–88 years), of a community-based cohort study from north-west London. The 10-item Geriatric Depression Scale was interviewer-administered during a clinic visit (depressive symptoms defined as a score of ⩾4 out of 10), with clinical data (adiposity, diabetes, cardiovascular disease, cognitive function) also collected. Sociodemographic, psychosocial, behavioural, disability, and medical history information was obtained by questionnaire.

**Results:**

Prevalence of depressive symptoms varied by ethnic group, affecting 9.7% of White European, 15.5% of South Asian, and 17.7% of Black Caribbean participants. Compared with White Europeans, South Asian and Black Caribbean participants were significantly more likely to have depressive symptoms (odds ratio 1.79, 95% confidence interval 1.24–2.58 and 1.80, 1.11–2.92, respectively). Adjustment for co-morbidities had most effect on the excess South Asian odds, and adjustment for socioeconomic position had most effect on the elevated Black Caribbean odds.

**Conclusions:**

Higher prevalence of depressive symptoms observed among South Asian people were attenuated after adjustment for physical health, whereas the Black Caribbean increased prevalence was most explained by socioeconomic disadvantage. It is important to understand the reasons for these ethnic differences to identify opportunities for interventions to address inequalities.

## Introduction

Depression is one of the leading causes of morbidity, healthcare use and loss of productivity worldwide (WHO, 2008). Its greater prevalence in older age is thought to be associated in part with chronic disease co-morbidity (Blazer *et al.* 1991), including cognitive dysfunction (Osborn *et al.* 2003), and in part with somatic symptoms and related mental disorders, such as anxiety (Yesavage *et al.* 1982).

People of South Asian and Black Caribbean origin form the largest and longest established ethnic minority populations in the UK. Most first-generation migration occurred in the 1950s and 60s, such that the majority are now of pensionable age. Variations in chronic disease prevalence have been identified (Smith *et al.* 2000), with both South Asian and Black Caribbean groups tending to have worse profiles (Fischbacher *et al.* 2007; Tillin *et al.* 2013). Despite marked ethnic group differences in risk factors for depression, including socioeconomic adversity, experience of discrimination, health behaviours, disability and chronic disease (Nazroo, 2003; Karlsen *et al.* 2005; Craig *et al.* 2006; Williams *et al.* 2011, 2012; Tillin *et al.* 2013), surprisingly few UK studies have characterized ethnic group variations in depression. Instead, the limited work has shown mixed results and comprised samples with wide or mid-life age ranges. Some studies have focused on elevated depression in South Asian women (Gask *et al.* 2011), and discordance with medication use (Hussain & Cochrane, 2002), yet in older samples, both elevated and equivalent rates of depression among South Asian people compared with Europeans have been reported (Silveira & Ebrahim, 1995; Lindesay *et al.* 1997). The sole comparative study of an older UK Black Caribbean sample reported no difference compared with the White population (Silveira & Ebrahim, 1995). Investigation of explanations for ethnic differences observed has been limited. Studies in the USA comparing the long established African American migrant population with US whites indicate excess or equivalent rates of depression (Husaini & Moore, 1990; Blazer *et al.* 1994; Simpson *et al.* 2007; Williams *et al.* 2007). Extrapolation to the UK context, however, may be unhelpful as UK Black Caribbeans are first-generation migrants, have different demographic and social characteristics, and are exposed to a different healthcare system; this argument would also apply to migrant populations to other settings outside the UK.

The aim of this paper was to compare depressive symptom prevalences between White European, South Asian and Black Caribbean older people from a community-based sample in West London, and to determine factors that might account for any observed differences, taking advantage of a longitudinal study with exposure measures obtained in both mid and later life. The primary hypothesis was that depressive symptoms would be more common in South Asian and Black Caribbean participants than Europeans. We hypothesized that excess chronic disease and socioeconomic disadvantage might explain elevated depressive symptoms in both ethnic minority groups compared with White Europeans. We also sought to investigate the extent to which exposure to other recognized risk factors for depression might potentially account for any prevalence differences observed. A secondary objective was to evaluate the extent to which the 10-item Geriatric Depression Scale (GDS; D'Ath *et al.* 1994) used in this study differed in performance between the three ethnic groups.

## Method

**Fig. 1.**
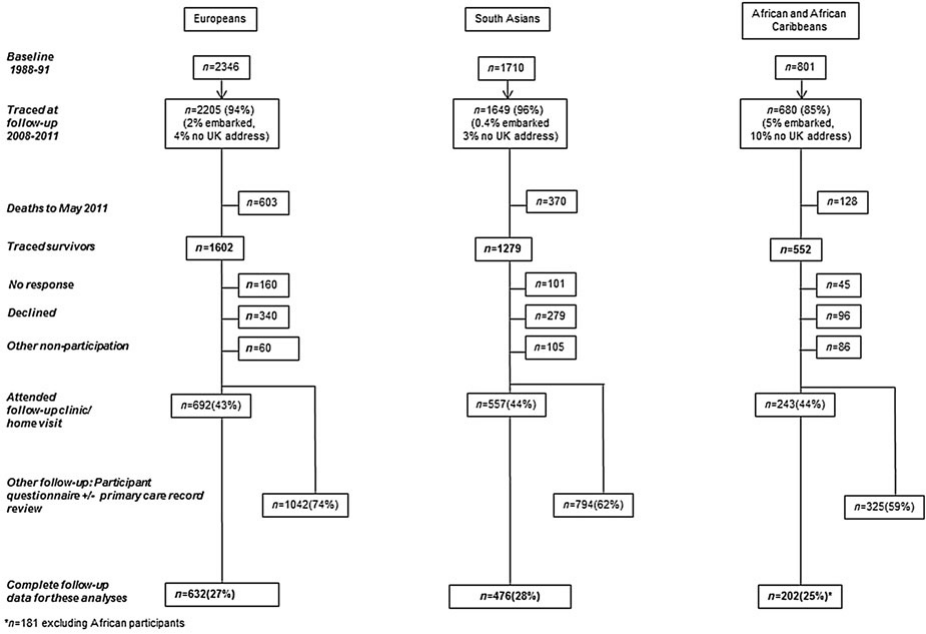
SABRE study flow diagram.

We analysed follow-up data from the Southall and Brent REvisited (SABRE) study, a community-based tri-ethnic cohort study of White European, South Asian and African and Black Caribbean individuals living in north-west London between 1988 and 1991 (Tillin *et al.* 2012) (see Fig. 1). Participants aged 40–69 (*n* = 4857; 2346 White, 1710 South Asian, 801 African and Black Caribbean) were randomly selected from age- and gender-stratified general practitioner lists and workplaces at baseline (1988–1991), and were followed up between 2008 and 2011. All South Asian, African and Black Caribbean participants were first-generation migrants. Since just 60 of the African and Black Caribbean participants originated from Africa, we have only included those of Black Caribbean origin in these analyses. Therefore, of the sample included here, all of the Black Caribbean people were born in the Caribbean, while the large majority (79%) of South Asian participants were born in the Indian subcontinent (69% India, 8% Pakistan, 2% Sri Lanka), with 15% born in East Africa; approximately half (53%) were of Punjabi Sikh origin. Of the White European sample included here, 87% were born in the UK and 15% were Irish-born. Ethnicity was interviewer-recorded based on parental origin and appearance and subsequently confirmed by participants. This method of self-assigned ethnicity is in keeping with the UK census categorization (http://www.ons.gov.uk/ons/guide-method/measuring-equality/equality/ethnic-nat-identity-religion/ethnic-group/index.html#1), modified to reflect the fact that our participants were all first-generation migrants, with no individuals of mixed ethnicity.

A 20-year follow-up investigation was conducted of all surviving participants (aged 58–88 years). Of the original sample, 93% were traced, of whom 3433 participants were alive at follow-up, with follow-up data available for 2161 participants (1042 European, 794 South Asian, and 325 Black Caribbean, equating to response rates of 65%, 62% and 59%, respectively). Detailed data on depressive symptoms and explanatory variables were collected from these participants who additionally attended clinic. Only participants with complete data on variables of interest were included in the main analyses [*n* = 1289: White European *n* = 632 (27%), South Asian *n* = 476 (28%), Black Caribbean *n* = 181 (25%)]. All participants gave written informed consent. Approval for the study at baseline was obtained from Ealing, Hounslow and Spelthorne, and University College London research ethics committees, and at follow-up from St Mary's Hospital Research Ethics Committee (ref.07/H0712/109).

### Assessments at baseline and follow-up

Table 1 displays the data collected at baseline and follow-up. Depressive symptoms were ascertained at follow-up only using the 10-item GDS (D'Ath *et al.* 1994), a widely used screening instrument administered by interviewer. The GDS has been specifically evaluated in previous research within UK ethnic groups (Abas *et al.* 1998) and a 4/10 cut-off was found to provide best performance against a diagnostic assessment (Stewart *et al.* 2001). Within the samples analysed here, the internal consistency for this scale was satisfactory (Cronbach's *α*: White European = 0.66; South Asian = 0.72; Black Caribbean = 0.69). At baseline, standard cardiometabolic assessments were performed (following an overnight fast), described elsewhere in detail (Table 1 and Tillin *et al.* 2012). A self-administered questionnaire included sociodemographic, behavioural and medical history items.

**Table 1.** Variables measured at baseline and follow-up

Variables	Scale	Measurement/categorization
Clinic visit – baseline (conducted by trained research staff)		
Waist circumference		Measurement taken halfway between costal margin and iliac crest
Hip circumference		Measurement taken over greater trochanter
Diabetes		Identified from medical record or participant recall of diagnosis
Coronary heart disease		Identified by data extracted from primary-care records (Tillin *et al.* 2012)
Stroke		Recorded according to primary-care data or participant report
Hypertension		Recorded according to primary-care data or participant report
Questionnaire – baseline (self-reported)		
Socioeconomic position	Manual labour	Dichotomized into *manual v. non-manual*
	Home tenure	Dichotomized into *own home v. do not own home*
Physical activity	Total weekly energy expended (MJ) in sport, walking and cycling, using questions and energy expenditure estimates (Durnin & Passmore, 1967)	Logarithmically transformed to accommodate negatively skewed data
Smoking	Smoking history	Dichotomized into *current v. ex/never smoker*
Clinic visit – follow-up (conducted by trained research staff)		
Depressive symptoms	10-item version of the Geriatric Depression Scale (GDS; D'Ath *et al.* 1994), well-validated in multi-ethnic samples (Abas *et al.* 1998)	Case-level depressive symptoms were defined as a score of ⩾4/10. The internal consistency for this scale was satisfactory (Cronbach's *α*: White European = 0.66; South Asian = 0.72; African Caribbean = 0.69)
Waist circumference		Measurement taken halfway between costal margin and iliac crest
Hip circumference		Measurement taken over greater trochanter
Diabetes		Identified from medical record, participant recall of diagnosis, or follow-up oral glucose tolerance test
Coronary heart disease		Identified by data extracted from primary-care records (Tillin *et al.* 2012)
Stroke		Recorded according to primary-care data or participant report
Hypertension		Recorded according to primary-care data or participant report
Cognitive function	Delayed word recall scores using the CERAD 10-word recall test (Morris *et al.* 1989)	Participants presented with 10 words on 3 occasions and were asked to recall them again after a delay of a few minutes. The number of correct words recalled after the delay was recorded
Questionnaire – follow-up (self-reported)		
Stressful life events	Life events scale assessed stressful life events over the preceding 2 years	List of 9 potentially stressful events; a dichotomous variable identified the presence or not of any stressful event over this period
Social contact		Measured by asking participants how many hours per week they spend with friends or family
Health state	EQ-5D (www.euroqol.org) (EQ-5D)	EQ-5D values transformed in a utility score using weights from the Measurement and Valuation of Health time trade-off (TTO) set of weights (Measurement and Valuation of Health Group, 1995; Dolan, 1997)
Physical activity	Total weekly energy expended (MJ) in sport, walking and cycling, using questions and energy expenditure estimates (Durnin & Passmore, 1967)	Logarithmically transformed to accommodate negatively skewed data
Smoking	Smoking history	Dichotomized into *current v. ex/never smoker*
Alcohol consumption	Regularity of consumption	Categorized into 5 groups (*never/special occasions only, once/twice a month, once/twice a week, four/five times a week, daily*)
Functional limitation		Impairment recorded if participants reported limitation with ⩾1 of following: (1) walking unaided without stopping and discomfort; (2) walking up and down a flight of 12 stairs without resting; (3) bending down to pick up a shoe from the floor

Participants were invited to complete a questionnaire and attend clinic at follow-up (Tillin *et al.* 2012). Questionnaire data were available for 60% of traced survivors from the original sample; however, since GDS data were collected during full-day clinic sessions only (conducted by trained research staff/nurses), response rates for this aspect of the study were lower (44% for White and Black Caribbean, 43% for South Asian participants).

### Statistical analysis

Age- and sex-adjusted group comparisons (reference category: White Europeans) were performed using analyses of covariance, logistic regression and Mann–Whitney *U* tests for continuous, categorical, and non-parametric (unadjusted) variables, respectively.

The main analyses used logistic regression to analyse ethnic group differences in depressive symptoms (reference category: White Europeans). We then wished to explore the impact of key factors, including socioeconomic position (SEP), chronic disease (either at baseline or follow-up) and functional limitation, in accounting for any ethnic differences observed in depressive symptoms. For example, we hypothesized that lower SEP in Black Caribbeans may determine excess levels of depressive symptoms; we therefore anticipated that adjustment for SEP would attenuate the Black Caribbean *v.* European odds ratio (OR). We first present each adjustment for individual groups of explanatory variables in turn, and then present a final multivariate model including all potential explanatory variables. Thus all models controlled for age and sex: model 1: age and sex only; model 2: SEP (manual labour, homeownership); model 3: stressful life events; model 4: baseline (4a) and follow-up (4b) health behaviours (smoking, physical activity); model 5: baseline (5a) and follow-up (5b) clinical factors/co-morbidities [waist circumference, diabetes, coronary heart disease (CHD), hypertension, stroke (follow-up only), functional limitation (follow-up only)]; model 6: cognitive function; model 7: depression/anxiety medication; model 8: adjustment for all covariates. The formula used to calculate the proportion of the association explained by adjustment was
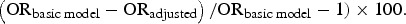


Sex by ethnicity interactions tested established sex differences in depressive symptoms (albeit less marked among older groups (Bebbington *et al.* 1998), but were found to be non-significant and not pursued further.

Because of missing data on alcohol consumption and social contact, these variables were excluded from the main analyses, and subgroup analyses were performed to check any impact on the main findings for ethnic group differences in depressive symptoms. Statistical analyses were performed using SPSS v. 20 (SPSS Inc., USA).

### GDS validation

To explore the equivalence of the GDS across the ethnic groups, differential item functioning (DIF) analyses were used to determine whether item bias was detected between ethnic groups (White European group as the reference category). For the identification of DIF, non-parametric tests within the DIFAS 4.0 program were used (Penfeld, 2007). The process used to measure DIF was the Mantel χ^2^ values (Mantel) and the Standardized Liu–Agresti Cumulative Common Log-odds ratio values (LOR *Z*). The EQ-5D was developed by the EuroQol group as a brief, self-completed measure of follow-up health state, rating five dimensions (mobility, self-care, usual activities, pain/discomfort, anxiety/depression) in three levels (no problems, some problems, extreme problems). A utility score was generated from the EQ-5D values using the Measurement and Valuation of Health time trade-off (TTO) set of weights (Measurement and Valuation of Health Group, 1995; Dolan, 1997). Taking advantage of its completion in this cohort, and in order to explore the construct validity of the GDS, bivariate correlations examined the relationship between the GDS and EQ-5D utility score and compared the strengths of this association between the three ethnic groups. Correlations were performed using SPSS v. 20.

## Results

Participants who attended follow-up clinics (therefore providing GDS data) differed from other survivors (follow-up responders without GDS data and non-responders) on baseline characteristics. People who did not attend clinics were younger (*p* < 0.001), more likely to be female (*p* = 0.006), have lower SEP (*p* < 0.001), and have poor self-rated health (*p* = 0.002). These differences applied equally across ethnic groups and clinic attendance rates were very similar across ethnic groups (44% European, 43% South Asian, 46% Black Caribbean).

### Comparison of characteristics (Table 2)

South Asian people were younger than White Europeans and Black Caribbean participants had a higher proportion of women than men. Both minority ethnic groups reported more manual occupation employment and less alcohol consumption compared with White Europeans. Although baseline and follow-up waist circumference was lower in South Asian than White European participants, prevalence of baseline and follow-up diabetes, follow-up CHD, and follow-up functional limitation were significantly higher. The Black Caribbean group showed increased prevalence of baseline and follow-up diabetes and follow-up functional limitation.

Overall, 13.0% of participants reported depressive symptoms, 9.7% of White European, 15.5% of South Asian (14.7% Indian, 21.1% Pakistani, 16.7% East African Asian), and 17.7% of Black Caribbean participants), a statistically significantly higher prevalence among both minority ethnic groups compared with their European counterparts (*p* = 0.004 and *p* = 0.001, respectively). The prevalence of depressive symptoms was higher in women than men, but this was not statistically significant (White European: 10.2% *v.* 9.5%, *p* = 0.93; South Asian: 20.3% *v.* 14.8%, *p* = 0.34; Black Caribbean: 22.6% *v.* 12.5%, *p* = 0.11, respectively).

**Table 2.** Characteristics of participants by ethnic group

	White European (*n* = 632)	South Asian (*n* = 476)	Black Caribbean (*n* = 181)
Age (years)	69.9 ± 6.3	68.7 ± 5.9*	70.7 ± 5.8*
Sex (% male)	78.3	86.6	48.6*
**Baseline**			
Manual labour: Manual	51.9	68.7*	66.9*
Home ownership: Own home	87.0	95.0*	70.2*
Smoking (%)			
Ex/never	79.1	89.9	84.0
Current	20.9	10.1*	16.0
Physical activity (hours/week)	10.8 (7.0–16.0)	9.3 (5.5–13.0)	11.0 (7.0–15.1)
Waist:hip ratio	0.90 ± 0.09	0.95 ± 0.08*	0.89 ± 0.08*
Waist circumference (cm)	88.0 ± 11.8	90.1 ± 9.4*	87.7 ± 10.3*
Diabetes (%)	2.5	13.4*	10.5*
Coronary heart disease (%)	5.9	5.9	8.9
Stroke (%)	0.2	0.2	1.0
Hypertension (%)	4.1	9.9*	22.7*
**Follow-up**			
Marital status (%)			
Married/cohabiting	67.7	85.9*	54.2*
Single	10.2	1.9	11.7
Divorced/separated	11.7	2.1	20.1
Widowed	10.4	10.2	14.0
Depressive symptoms (%)	9.7	15.5*	17.7*
Antidepressant/anxiolytic medication (% taking)	4.7	3.2	5.0
Stressful life events (% reporting any)	55.4	49.2*	59.7
Social contact (hours/week)	9.0 (4.0–20.0)	8.6 (3.9–20.0)	6.5 (3.0–15.8)*
Smoking (%)			
Ex/never	92.7	95.8	95.6
Current	7.3	4.2*	4.4
Physical activity (hours/week)	3.8 (1.8–5.8)	3.8 (1.8–4.7)*	3.8 (1.8–4.7)*
Alcohol intake (%)			
Daily	21.8	20.1	6.3
Four/five times a week	21.6	16.1	8.9
Once/twice a week	24.7	27.5	22.2
Once/twice a month	11.7	8.7	13.9
Never/special occasions	20.1	27.5*	48.7*
Waist:hip ratio	0.97 ± 0.07	1.00 ± 0.07*	0.96 ± 0.08*
Waist circumference (cm)	100.1 ± 13.3	97.8 ± 10.2*	97.7 ± 12.1
Diabetes (%)	19.3	42.1*	39.2*
Coronary heart disease (%)	20.4	34.5*	13.2
Stroke (%)	2.7	2.1	5.0
Hypertension (%)	55.2	75.2*	77.9*
Functional limitation (%)	14.7	32.8*	29.8*
Cognitive function (no. of recalled words)	6.1 ± 2.0	5.8 ± 2.0*	5.1 ± 2.1*

Data presented are unadjusted means (s.d.) and %, with exception of social contact and physical activity, presented as medians (interquartile range), due to skewed data.

* *p* < 0.05 for age- and sex-adjusted group differences with White European as the reference category.

### Ethnic group differences in depressive symptoms between South Asian and White European participants (Table 3)

South Asian participants had significantly elevated odds [OR 1.79, 95% confidence interval (CI) 1.24–2.58] of having depressive symptoms compared with White Europeans (model 1). Socioeconomic, stressful life event (follow-up), and behavioural characteristics (models 2–4) had little impact on odds ratios. While baseline clinical factors/co-morbidities did not affect the odds ratios (model 5a), the follow-up measures of these variables accounted for most of the elevated odds of depressive symptoms, 65% (model 5b), reducing the ethnic difference to non-significance. This was explored further and functional limitation alone accounted for an additional 67% of the excess odds, after adjustment for other baseline and follow-up co-morbidities. Cognitive function and antidepressant/anxiolytic medication use (models 6–7) did not affect the differences observed. In the fully adjusted model 8, the South Asian odds remained significantly elevated compared with White Europeans.

**Table 3.** Odds of having depressive symptoms for South Asian and Black Caribbean participants (compared with White European participants)

	South Asian	Black Caribbean
	OR (95% CI)	OR (95% CI)
Model 1: Adjusted for age and sex	1.79 (1.24–2.58)	1.80 (1.11–2.92)
Model 2: Adjusted for age, sex, and socioeconomic position	1.80 (1.23–2.63)	1.46 (0.89–2.39)
Model 3: Adjusted for age, sex, and stressful life events	1.86 (1.29–2.69)	1.76 (1.08–2.87)
Model 4a: Adjusted for age, sex, and baseline health behaviours	1.86 (1.26–2.74)	1.93 (1.18–3.13)
Model 4b: Adjusted for age, sex, and follow-up health behaviours	1.71 (1.17–2.49)	1.88 (1.15–3.10)
Model 5a: Adjusted for age, sex, and baseline clinical risk factors/co-morbidities	1.79 (1.22–2.60)	1.91 (1.15–3.16)
Model 5b: Adjusted for age, sex, and follow-up clinical risk factors/co-morbidities	1.28 (0.84–1.94)	1.81 (1.07–3.04)
Model 6: Adjusted for age, sex, and cognitive function	1.71 (1.18–2.47)	1.62 (0.99–2.64)
Model 7: Adjusted for age, sex, and depression/anxiety medication	1.85 (1.28–2.68)	1.83 (1.12–2.98)
Model 8: Adjustment for all covariates	1.63 (1.02–2.60)	1.98 (1.12–3.49)

OR, Odds ratio; CI, confidence interval.

Reference category: White European. Model 2 included adjustment for manual occupation and home ownership; model 4a included adjustment for baseline smoking and physical activity; model 4b included adjustment for follow-up smoking and physical activity; model 5a included adjustment for baseline waist circumference, diabetes, coronary heart disease, and hypertension; model 5b included adjustment for follow-up waist circumference, diabetes, coronary heart disease, stroke, hypertension, and functional limitation.

### Ethnic group differences in depressive symptoms between Black Caribbean and White European participants (Table 3)

Black Caribbean participants were significantly more likely to report depressive symptoms compared with White Europeans (OR 1.80, 95% CI 1.11–2.92). Socioeconomic disadvantage (model 2) explained a substantial proportion of the elevated odds observed (43%), while psychosocial, behavioural, and clinical factors/co-morbidity factors had only marginal effects on the excess depressive symptoms in this group (models 3–5). Cognitive function (model 6) accounted for 23% of the elevated odds of depressive symptoms among Black Caribbean participants, while antidepressant/anxiolytic medication use did not affect this differential (model 7). Full adjustment (model 8) did not attenuate the elevated odds ratio, thus Black Caribbeans were nearly twice as likely to report depressive symptoms even when other factors were taken into account.

Alcohol intake and social contact were included in separate versions of model 8 and did not qualitatively affect the findings of the main analyses.

### GDS validation

No GDS items elicited Mantel scores >6.63 in either the South Asian or Black Caribbean responses, compared with those of White Europeans [the maximum value above which group differences in response to the studied items are indicated (Penfeld, 2007)]. Similarly, none of the LOR *Z* values were greater than 2.0 or less than −2.0 (reflecting DIF effect size). Therefore, no DIF was identified for the GDS. The correlations for GDS and EQ-5D utility scores were comparable across ethnic groups [*r* = −0.38 for White European (*p* < 0.001), *r* = −0.35 for South Asian (*p* < 0.001), *r* = −0.30 for Black Caribbean participants (*p* < 0.001)].

Interestingly, one item referring to whether people thought that most other people were better off than them was more heavily endorsed by the South Asian and Black Caribbean participants, compared with the White Europeans (19% of South Asian, 20% of Black Caribbean and 5% of White European participants responded positively to this question). Removal of this item did not significantly influence the results.

## Discussion

Older first-generation migrants of South Asian and Black Caribbean origin to the UK were nearly twice as likely to report depressive symptoms as their European origin counterparts. Different factors appeared to account for this excess. In South Asians, these factors included a greater degree of later-life chronic disease, while in Black Caribbeans the key contributor appeared to be socioeconomic disadvantage.

### Strengths and limitations

Depression is highly prevalent in older populations (Osborn *et al.* 2003). This study provides the largest comparison of depressive symptoms among the UK's three most populous ethnic groups within this age range, recruited from the general population. Other major strengths are the range of possible longitudinal mid- and late-life factors assessed, and the use of a depression measure with evidence of cross-cultural validity designed specifically for use in older people. However, there are some limitations. The primary outcome only ascertained symptomatology on a relatively brief screening instrument that was not a diagnostic instrument; nonetheless, this instrument strongly predicts subsequent depressive symptoms (Vinkers *et al.* 2004). The attrition during follow-up and missing data mean that our sample may be subject to selection bias. We compared baseline characteristics of those with and without GDS data, and we showed that healthier individuals and those with higher SEP were more likely to re-attend. This differential response applied equally to all ethnic groups; thus the observed ethnic group variations in depressive symptoms are unlikely to have been influenced by this bias. The study's cross-sectional ascertainment of depressive symptoms at follow-up preclude conclusions around direction of causality [because symptomatology in mid-life (baseline) and the intervening period were not recorded] and does not capture the episodic nature of depression (although incorporation of data on antidepressant use may help to some limited extent). While adjustment for stressful life events did not markedly alter the ethnic difference in reported depressive symptoms for either South Asians or Black Caribbeans, we acknowledge that these events may be differently remembered, or have different impacts by ethnicity. Factors included in our models may act as causal determinants, confounders or consequences of depressive symptoms and it is possible, for example, that adjustment for physical disease represented an over-adjustment if this was a consequence of depressive symptoms. Furthermore, we could not measure all factors that may account for ethnic differences in depressive symptoms.

### Comparison with previous studies

An East London study found substantially elevated depression prevalence among Bengali compared with Somali and White British older participants, citing, but not testing, socioeconomic variables and social support as potential explanations (Silveira & Ebrahim, 1998). By contrast, a comparison of Gujarati and White people aged >65 years from Leicester showed no ethnic differences in depression (Lindesay *et al.* 1997). Data from the USA indicate an excess depression prevalence among Black American compared with White participants (Skarupski *et al.* 2005), which was only partially attenuated after adjustment for socioeconomic characteristics, although another study found no significant difference in the same scores using the same instrument between African American and White older adults (Blazer *et al.* 1998). However, generalizing between ethnic groups in different countries is limited because of substantial differences in SEP, social integration and migration history.

### Depressive symptoms in South Asians

The excess depressive symptomatology among South Asian people observed here was largely attenuated by functional limitation adjustment (Table 3 model 5b), suggesting that the elevated disability risk among South Asian people (Williams *et al.* 2012) may contribute to higher depressive symptoms. Differences in body composition and chronic disease are well established between UK White and South Asian groups (Bhopal *et al.* 1999) but did not appear to account for the difference in depressive symptoms, beyond the impact of disability. Although subgroup analyses were not possible, unadjusted prevalences of depressive symptoms were higher among all South Asian subgroups than White Europeans, consistent with some earlier research (Williams *et al.* 2010), but not others (Nazroo, 1997). The predominant subgroup in this sample was Punjabi Sikh (53%), who tend to occupy a higher SEP than other subgroups (Williams *et al.* 2010), and our results may therefore underestimate the prevalence of depressive symptoms in South Asian communities as a whole.

### Depressive symptoms in Black Caribbeans

Adjustment for socioeconomic measures and cognitive function attenuated the excess depressive symptoms in Black Caribbean participants. UK Black Caribbean people have often experienced socioeconomic disadvantage (Smith *et al.* 2000), which is a well-recognized risk factor for depression (Katon *et al.* 2004). Other socioeconomic characteristics, such as wealth, were not taken into account and therefore may have contributed further to the excess prevalence of depressive symptoms in this group. However, there is a surprising dearth of comparable data in this group and further work is needed to confirm our findings.

### Validity of depression questionnaire

It is important to bear in mind potential differences in interpretation when comparing depression measurements between ethnic groups (Nazroo, 1997), and qualitative research has, for example, found significant variations in mental distress language and presentation between White and Pakistani people, explaining different patterns of help-seeking and health outcomes (Mallinson & Popay, 2007). However, our analyses of GDS performance and cultural equivalence did not suggest consistent differences between the three ethnic groups in the probability of giving a certain GDS response, suggesting similar interpretation of questions across groups, and supporting previous validation of this instrument (Abas *et al.* 1998). In addition, there was no meaningful difference in the correlation between GDS and EQ-5D scores, although it should be borne in mind that these analyses, although supportive, represented an opportunistic use of available data and the cohort study was not formally designed to assess the validity of the GDS. Our assessment of the GDS validity did not test all components of validity that are necessary to comprehensively evaluate the cultural equivalence of this instrument (Flaherty *et al.* 1988).

### Unmeasured explanations

High levels of endorsement of the GDS item associated with feelings of relative disadvantage by the South Asian and Black Caribbean participants were found. This may reflect experiences of discrimination (Smith *et al.* 2000) but additional focused research would be needed to clarify this. Indeed, it is important to bear in mind that there are other psychosocial and cultural explanations that were insufficiently measured. Racial discrimination is a risk factor for depression (Karlsen *et al.* 2005), with experience of interpersonal and institutionalized racism shown to be particularly associated with worse mental health (Karlsen *et al.* 2005). Research exploring depression in UK South Asian populations has implicated the role of culture conflict as a potential cause (Hussain & Cochrane, 2002; Bhugra, 2003; Gask *et al.* 2011), possibly particularly in first-generation migrants and South Asian women for whom acculturation levels can be lower (Ghuman, 2000). Family conflict as younger generations assimilate has also been associated with depression among older South Asian people (Sonuga-Barke & Mistry, 2000). Since all minority ethnic participants were first-generation migrants, the differences in depressive symptoms may reflect issues associated with migration (Bhugra, 2003), rather than ethnicity. Bhugra's migration and depression review highlights the importance of social support and isolation resulting from migration (Bhugra, 2003), and isolation was seen as contributing to depression onset and maintenance among depressed Pakistani women in North-West England (Gask *et al.* 2011).

This study's findings present important implications for depression prevention and treatment. Economic evaluation of interventions to reduce physical disease throughout mid- and late-life should acknowledge the possible beneficial effects these may also have on subsequent mental health and its sequelae. Social policy interventions should also recognize the benefits to mental health inequalities. Limited evidence suggests that receipt of medication for depression is less common in ethnic minority groups, perhaps in part reflecting the difficulty in diagnosis or cultural differences in acceptability. Our findings require replication and their generalizability needs to be clarified – both to other ethnic groups, and to subsequent generations whose health status, behaviours, socioeconomic status and acculturation may differ. It is important that general and mental health practitioners are aware of these elevated depressive symptom prevalences among certain ethnic groups, and are able to recognize other factors, such as co-morbidities, that might identify individuals at ‘high risk’. Equitable access to mental health services with effective and culturally appropriate treatment must be available for vulnerable groups.
